# Ultrastrong and Tough Urushiol-Based Ionic Conductive Double Network Hydrogels as Flexible Strain Sensors

**DOI:** 10.3390/polym15153219

**Published:** 2023-07-28

**Authors:** Fengcai Lin, Yiwen Zhu, Zixuan You, Wenyan Li, Jipeng Chen, Xiaoxiao Zheng, Guocai Zheng, Zifan Song, Xinda You, Yanlian Xu

**Affiliations:** 1 Fujian Engineering and Research Center of New Chinese Lacquer Materials, College of Materials and Chemical Engineering, Minjiang University, Fuzhou 350108, China; fengcailin@mju.edu.cn (F.L.); yiwenzhu@stu.mju.edu.cn (Y.Z.); youzixuan@stu.mju.edu.cn (Z.Y.); 3202314103@stu.mju.edu.cn (W.L.); jpchen@mju.edu.cn (J.C.); xxzheng@mju.edu.cn (X.Z.); 2231@mju.edu.cn (G.Z.); 2 College of Material Engineering, Fujian Agriculture and Forestry University, Fuzhou 350108, China; 3211038028@fafu.edu.cn

**Keywords:** urushiol, ultrastrong, double-network hydrogel, ionic conduction, sensor

## Abstract

Ionic conductive hydrogels have attracted increasing research interest in flexible electronics. However, the limited resilience and poor fatigue resistance of current ionic hydrogels significantly restrict their practical application. Herein, an urushiol-based ionic conductive double network hydrogel (PU/PVA-Li) was developed by one-pot thermal initiation polymerization assisted with freeze–thaw cycling and subsequent LiCl soaking. Such a PU/PVA-Li hydrogel comprises a primary network of covalently crosslinked polyurushiol (PU) and a secondary network formed by physically crosslinked poly(vinyl alcohol) (PVA) through crystalline regions. The obtained PU/PVA-Li hydrogel demonstrates exceptional mechanical properties, including ultrahigh strength (up to 3.4 MPa), remarkable toughness (up to 1868.6 kJ/m^3^), and outstanding fatigue resistance, which can be attributed to the synergistic effect of the interpenetrating network structure and dynamic physical interactions between PU and PVA chains. Moreover, the incorporation of LiCl into the hydrogels induces polymer chain contraction via ionic coordination, further enhancing their mechanical strength and resilience, which also impart exceptional ionic conductivity (2.62 mS/m) to the hydrogels. Based on these excellent characteristics of PU/PVA-Li hydrogel, a high-performance flexible strain sensor is developed, which exhibits high sensitivity, excellent stability, and reliability. This PU/PVA-Li hydrogel sensor can be effectively utilized as a wearable electronic device for monitoring various human joint movements. This PU/PVA-Li hydrogel sensor could also demonstrate its great potential in information encryption and decryption through Morse code. This work provides a facile strategy for designing versatile, ultrastrong, and tough ionic conductive hydrogels using sustainable natural extracts and biocompatible polymers. The developed hydrogels hold great potential as promising candidate materials for future flexible intelligent electronics.

## 1. Introduction

In recent years, flexible, stretchable, and wearable electronic intelligent devices have drawn significant attention for applications in sensors, actuators, and human–machine interfaces [1,2,3]. These electronic devices possess the ability to detect alterations in external stimuli in real time and accurately convert them into electrical signals with a high degree of precision and sensitivity. Traditional flexible devices have been prepared by incorporating metal nanoparticles (silver or copper nanowire [4,5], silver or gold nanoparticles [6,7]), and carbon nanomaterials (carbon nanotube [8], graphene [9], and MXene [10]) into elastomers substrates. However, the unsatisfactory compliance and stretchability of these devices, which make it difficult for them to match human joints, have seriously impeded their practical applications. Hydrogels, consisting of three-dimensional (3D) polymeric networks with large amounts of solvent, have been considered promising candidates to solve the above problems. The solvents present in the matrices impart to the hydrogel good electrolyte-dissolving capability. The dissolved electrolyte can be transported into the porous structure of the hydrogel, which is saturated with solvent. Consequently, ionic conductive hydrogel was obtained and has subsequently been utilized for wearable electronic intelligent devices [11].

Ionic hydrogels with excellent flexibility, stretchability, and sensitivity are promising candidates for flexible sensing systems and wearable electronic devices [12]. The distinctive 3D network structure of ionic conductive hydrogels provides efficient channels for ion transporting, enabling designable functionality [13]. Poly(vinyl alcohol) (PVA) is a versatile linear polymer with a regular structure and a high density of hydroxyl groups, making it suitable for the construction of ionic conductive hydrogels. Through the process of cyclic freezing–thawing, the hydroxyl groups on PVA polymer chains can form hydrogen-bonded crystalline structures, which are commonly utilized to construct multifunctional ionic hydrogels [11]. However, PVA ionic hydrogels prepared by cyclic freezing–thawing methods usually suffer from poor mechanical performance due to the lack of reliable energy dissipation mechanisms, seriously restricting their applications in flexible electronic devices. Different strategies have been explored to synthesize mechanically robust PVA ionic conductive hydrogels, including double networks [14,15], nanocomposite enhancement [16], and macromolecular microsphere compositing [17]. Among these methods, constructing a double network (DN) structure within PVA ionic conductive hydrogels is an effective approach to expand the energy dissipation mechanism of hydrogels. For instance, Zhou et al. [14] synthesized a polyacrylamide/PVA/xanthan gum/ZnCl_2_ ionic conductive DN hydrogel via one-pot in situ polymerization and a subsequent cyclic freezing–drying method, and they found that the synergistic effect of intermolecular chemical covalent crosslinking and physical crosslinking were responsible for the enhanced mechanical strength of hydrogels. Liu et al. [18] developed a “freezing–drying–swelling” cycle strategy to prepare a PVA/polyacrylic acid (PAA)/CaCl_2_ ionic conductive DN hydrogel. By incorporating the PVA crystalline domains and H-bonds formed during the freezing–drying process, along with additional H-bonds provided by the second PAA network, the resulting hydrogel exhibited an impressive ultimate tensile stress of 1.1 MPa and remarkable stretchability of 648% strain. However, the above PVA hydrogels based on DN strategy still suffered from low crosslinking density, leading to a relatively loose structure that weakens the physical interactions between the two networks. Hence, the prepared PVA ionic hydrogels still demonstrated limited tensile strength (<2 MPa) and fatigue resistance, restricting their practical applications. Additionally, some of the current PVA dual network ionic conductive hydrogels are constructed using petroleum-based polymers, which may contain potential harmful monomers (such as acrylamide or acrylic acid) or residual crosslinking agents within the hydrogel. Therefore, developing a PVA dual network ionic hydrogel that combines good mechanical properties, excellent resilience, and reliable sensitivity based on the principles of green and sustainable development remains a challenge.

**Scheme 1:**
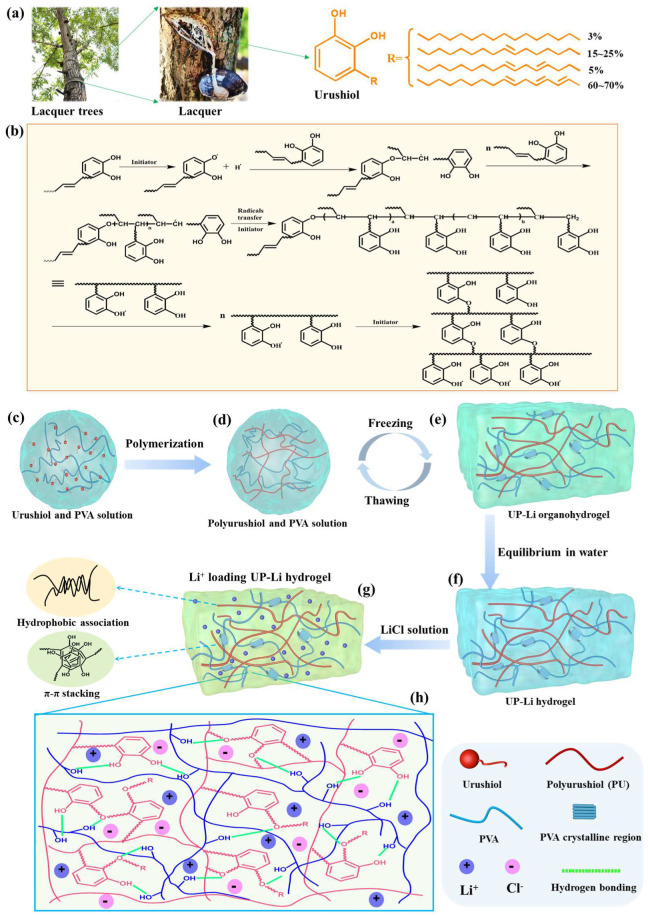
(**a**) Chemical structure of urushiol. (**b**) The polymerization mechanism of urushiol [25,26]. (**c**–**h**) The synthesis procedure of PU/PVA-Li hydrogels and their potential mechanism of formation.

Urushiol, derived from the lacquer tree, is a kind of natural sustainable resource consisting of a benzene ring, catechol groups, and an unsaturated C15–17 aliphatic side chain with 1–3 double bonds [19] (Scheme 1a). The presence of an aromatic ring and a long alkenyl side chain impart to urushiol both rigidity and flexibility [20], which make it perhaps an ideal candidate to enhance the mechanical strength and toughness of PVA ionic hydrogels. Triggered by initiator, reactions between double bonds and catechol groups occur; therefore, urushiol will self-polymerize to form a highly crosslinked dense 3D network structure [19,21] (Scheme 1b). The obtained polyurushiol (PU) exhibits many excellent properties, including high mechanical strength, thermal and chemical stability, and aging resistance, and it has been widely used as a high-performance adhesive or coating material since the period of ancient China more than 7000 years ago [19,22]. Based on the excellent properties of PU, we hypothesize that the introduction of PU into PVA ionic conductive hydrogels may significantly improve their toughness and fatigue resistance, thereby promoting their extensive applications in the field of flexible electronics.

Herein, an urushiol-based ionic conductive double-network hydrogel (PU/PVA-Li) was developed by integrating a covalently crosslinked PU three-dimensional network with a physically crosslinked PVA network, followed by loading of LiCl. Our PU/PVA-Li hydrogel demonstrates exceptional mechanical properties, including ultrahigh strength, remarkable toughness, and excellent fatigue resistance, attributed to the interpenetrating network structure of PU and PVA chains. When the PU/PVA-Li hydrogel undergoes external loading, the easy destruction of the physical interaction (inter/intra-molecular hydrogen bonding, hydrophobic associations, π-π stacking) between PU and PVA chains acts as the sacrificial bond to readily absorb and dissipate energy, while the highly crosslinked PU networks maintain the configuration of the hydrogel without fracturing. In addition, the incorporation of LiCl into the hydrogels not only results in polymer chain contraction through ionic coordination [23,24], thereby improving mechanical strength and resilience, but also confers remarkable ionic conductivity upon the hydrogels. Based on these features, the PU/PVA-Li hydrogel was assembled as a flexible strain sensor for monitoring various human joint movements. Meanwhile, our PU/PVA-Li hydrogel sensor can be further used as a signal transmitter for information encryption and decryption through Morse code. Therefore, this work provides a promising approach for designing PVA ionic conductive hydrogels with outstanding mechanical properties using sustainable natural resources. The synthesized PU/PVA-Li hydrogel exhibits significant potential for diverse applications in flexible electronic intelligent devices.

## 2. Materials and Methods

### 2.1. Materials

Urushiol was extracted from lacquer obtained from Maoba, Hubei Province, China, using the ethanol extraction method described in the literature [20]. Poly(vinyl alcohol) (PVA, DS = 1750 ± 50), dimethyl sulfoxide (DMSO, more than 99.0% purity), potassium persulfate (K_2_S_2_O_8_), and lithium chloride (LiCl) were procured from Sinopharm Chemical Reagent Co., Ltd. (Beijing, China). The chemicals were employed without additional purification, and distilled water was utilized for the formulation of aqueous solutions.

### 2.2. Synthesis of PU/PVA-Li Hydrogels

Ultrastrong, tough, and ionic conductive polyurushiol (PU)/poly(vinyl alcohol) (PVA) (PU/PVA-Li) hydrogels were constructed using a two-step method. First, PU/PVA double-network (DN) hydrogel was prepared. A PU/PVA homogeneous solution was obtained by dissolving 4.5 g of PVA, 3.15 g of urushiol, and 0.0076 g of K_2_S_2_O_8_ in 25.5 g of DMSO, followed by heating to 120 °C with vigorous stirring for 3 h. Once the air bubbles were eliminated through ultrasonication, the mixtures were poured into the mold, frozen at 25 °C for 12 h, and subsequently thawed to room temperature over a period of 6 h. The freezing–thawing cycle was repeated three times, resulting in the formation of the PU/PVA DN hydrogels. The obtained PU/PVA hydrogels were purified by immersing them in a substantial quantity of pure water for 72 h to completely remove the DMSO and initiator. Subsequently, the PU/PVA DN hydrogels with a thickness of 1.0 mm were immersed in varying concentrations of LiCl solution (0.0 M, 0.4 M, 0.5 M, 0.6 M, 0.7 M, 0.8 M) for 24 h to create ionic conductive PU/PVA DN hydrogels. For comparison, pure PVA hydrogels were also prepared and denoted as PVA-gel. The preparation process for PU/PVA-Li hydrogels is illustrated in Scheme 1.

### 2.3. Structural Characterization

The FTIR spectra of the PU/PVA-Li hydrogels were obtained via a NI-COLET 380 FTIR spectrometer (Thermo Electron Instruments Co., Ltd., Waltham, MA, USA). The samples were scanned in the range of 500–4000 cm^−1^ with 32 scans. The internal structure of the PU/PVA-Li hydrogels was studied using a scanning electron microscope (SEM, SU8010, Hitachi, Japan) at an acceleration voltage of 40 kV. Prior to observation, the hydrogels were freeze-dried and coated with a layer of gold. Thermal gravimetric analysis (TGA) of the hydrogels was conducted using a thermal gravimetric analyzer (NETZSCH STA 449 F3 Jupiter, Netzsch Gertebau GmbH, Bavaria, Germany). The samples underwent heating from room temperature to 600 °C at a heating rate of 10 °C/min under a N_2_ atmosphere with a flow rate of 30 mL/min.

### 2.4. Mechanical Measurement

The mechanical properties of the PU/PVA-Li hydrogels were assessed at ambient temperature utilizing a universal material testing machine (ETM520C, WANCE, Shenzhen, China). During the experimental procedures of tensile and cyclic loading tests, the hydrogel specimen was meticulously shaped into a dumbbell configuration and subsequently exposed to a controlled tensile rate of 50 mm/min. The 50 continuous tensile–relaxation cycles were conducted using a load–unload methodology, employing a stretching rate of 50 mm/min and stretching strains of 100%, 200%, 300%, and 500%. Stress–strain curves were utilized to determine the elastic modulus, toughness, and dissipated energy of the specimens. To prevent water evaporation during the experiment, a thin layer of silicon oil was applied onto the hydrogel surface.

### 2.5. Electrical Behavior Measurements

An LCR meter (TH2830, Changzhou Tonghui Electronics Co., LTD, Changzhou, China) was used to measure the conductivity of the PU/PVA-Li hydrogels at room temperature. The hydrogels’ conductivity (δ) was determined by employing the formula δ = d/RS, where d represents the length, R stands for the resistance, and S denotes the cross-sectional area. To assess the changes in resistance during the process of stretching, the samples were firmly affixed to a universal testing machine employing a stretching rate of 50 mm/min. The resistance values were subsequently documented utilizing an LCR meter.

### 2.6. Hydrogel Based Flexible Sensor Assembling

A wearable hydrogel sensor composed of PU/PVA-Li was constructed by enclosing the hydrogel (20 mm × 5 mm × 1 mm) between two VHB tapes (4905, 3 M) and connecting it to two metallic electrodes (as depicted in Figure 5i). The electrical signals of the PU/PVA-Li hydrogel sensor were monitored in real time using an LCR meter (TH2830, China).

## 3. Results and Discussion

### 3.1. Preparation and Structure of PU/PVA-Li Hydrogels

**Figure 1:**
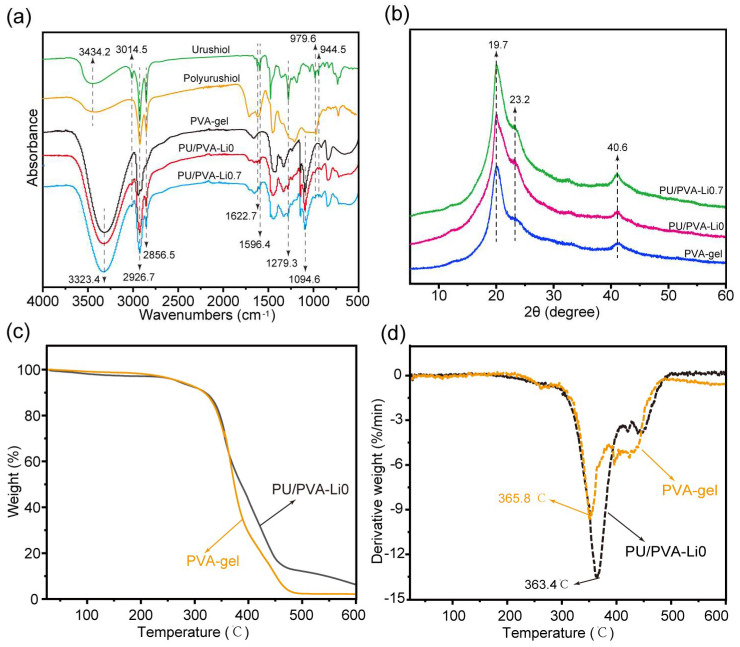
(**a**) FTIR spectra of urushiol, polyurushiol (obtained from the polymerization of urushiol in DMSO solution), PVA-gel, PU/PVA-Li0, and PU/PVA-Li0.7. (**b**) XRD patterns of PVA-gel, PU/PVA-Li0, and PU/PVA-Li0.7. TG (**c**) and DTG (**d**) curves of PVA-gel and PU/PVA-Li0.

As illustrated in Scheme 1a, urushiol is a natural catechol derivative with a long alkyl side chain containing 1–3 unsaturated double bonds, which can carry out free-radical polymerization. As shown in Figure 1b, during the process of polymerization, the decomposition of hydroxyl groups in urushiol generates a phenoxy radical, which serves as an initiator for the formation of -C=C- bonds on the side chains of the phenyl ring, thereby initiating chain propagation and leading to the formation of a two-dimensional polymer [25]. Concurrently, the unsaturated groups on the side chains, initiated by the radicals, undergo further polymerization, resulting in the formation of a three-dimensional polymer structure. The general procedure to synthesis PU/PVA-Li interpenetrating network hydrogels is illustrated in Scheme 1b–g. First, urushiol, initiator (K_2_S_2_O_8_), and PVA are dissolved in DMSO and heated to complete the polymerization of urushiol, obtaining homogeneous polyurushiol (PU)/PVA solution (Scheme 1b,c). After polymerization, the obtained PU with a three-dimensional polymer structure serves as the first network for the PU/PVA-Li hydrogel. The PU/PVA DN hydrogel was synthesized using a cyclic freezing–thawing method, employing a PU and PVA homogeneous solution (Scheme 1c,d). After undergoing three freezing-thawing cycles, the PVA chains formed crystalline domains that acted as knots for the second physically crosslinked network within PU/PVA hydrogel. Then, the obtained PU/PVA hydrogels were purified by soaking in large amounts of pure water for 72 h to remove the DMSO and initiator completely (Scheme 1e,f). During this process, the strong hydrophobicity of the lengthy alkyl chains present in the side chains of urushiol facilitated the formation of hydrophobic associations within the PU/PVA-Li hydrogel network of PU, while the adjacent benzene groups of PU contributed to a π–π stacking interaction (Scheme 1g). These hydrophobic moieties serve as crucial physical crosslinking points, significantly improving the mechanical characteristics of the hydrogel. After purification, the purified PU/PVA hydrogel was further soaked in LiCl aqueous solution of a desired concentration to load LiCl and form ionic conductive PU/PVA-Liɑ DN hydrogels (where ɑ is the concentration of LiCl) (Scheme 1f,g). Moreover, the subsequent introduction of Li^+^ ions, which can coordinate with phenolic hydroxyl groups, facilitates the generation of hydrophobic interactions within the polymer network, leading to further enhancement of the mechanical properties of the composite hydrogel. As illustrated in Scheme 1g, the PU/PVA-Li hydrogel consists of a covalently crosslinked PU and hydrogen-bonded PVA network. Simultaneously, the two interpenetrating networks interact with each other through intermolecular H-bonding between the hydroxyl groups of PU and PVA (Scheme 1g). In this case, the covalent PU three-dimensional network provides elasticity, while the hydrogen-bonded PVA crystallites function as stable and reversible physical crosslinkers during mechanical deformation, leading to PU/PVA-Li hydrogels with outstanding stretchability and toughness.

Figure 1a shows the FTIR spectra of the urushiol, PVA-gel, PU/PVA-Li0, and PU/PVA-Li0.7. As shown in Figure 1a, the spectrum of urushiol shows some characteristic peaks at 3434.2 cm^−1^ (hydroxyl group stretching vibration), 3014.5 cm^−1^ (=C-H stretching vibration), 979.6 cm^−1^ and 944.6 cm^−1^ (the bending vibration of triene structures in long side chains) [22,27]. However, these absorption peaks almost disappeared in the spectrum of polyurushiol, indicating that urushiol was successfully polymerized. In the spectra of PU/PVA-Li0, the feature absorption peaks at 2926.7 cm^−1^, 2856.5 cm^−1^, 1622.7 cm^−1^ (C-H stretching vibration), and 1596.4 cm^−1^ (benzene ring stretching vibration) confirmed the existence of PU in the PU/PVA-Li0 hydrogel [28]. Meanwhile, the PU/PVA-Li0 hydrogel also displayed several characteristic peaks of PVA-gel, including 3323.4 cm^−1^ (OH stretching vibration), 2926.7 cm^−1^(C-H stretching vibration), and 1094.6 cm^−1^ (C-O stretching vibration) [29], indicating the successful construction of PU/PVA-Li interpenetrating network hydrogels. Furthermore, comparing the FTIR spectra of PU/PVA-Li0.7 and PU/PVA-Li0, no new absorption peaks or notable distinctions were observed for PU/PVA-Li0.7, indicating that the introduction of LiCl did not impact the chemical structure of the hydrogel. In addition, the internal structure of the composite hydrogels was further analyzed by XRD analysis. As shown in Figure 1b, neat PVA-gel exhibited a prominent crystalline reflection at 2*θ* = 19.7°, specifically corresponding to the (101) plane, which is a characteristic crystalline peak of PVA [30]. The additional two minor diffraction reflections have been attributed to PVA’s (200) and (102) crystal planes [31]. In the patterns of PU/PVA-Li0 and PU/PVA-Li0.7, the characteristic peaks at 2*θ* = 19.7°, 23.2°, and 40.6° remain almost unchanged, suggesting that the introduction of PU and the subsequent LiCl loading process did not impact the crystalline structure of PVA-gel. Within the PU/PVA-Li hydrogel matrix, LiCl is present as hydrated lithium ions and hydrated chloride ions, thereby maintaining the crystalline structure of PVA without any notable effects. 

Representative TG and DTG curves of PVA-gel and PU\PVA-Li0 are demonstrated in Figure 1c,d. As depicted in Figure 1c, both PVA-gel and PU\PVA-Li0 showed an initial weigh loss at 25–200 °C, which could be attributed to the evaporation of hydrogen-bound water [32]. In the high-temperature range, PVA-gel and PU/PVA-Li0 hydrogel exhibited different patterns of weight loss. It is worth noting that the PU\PVA-Li0 DN hydrogel demonstrated an increase in initial thermal degradation temperature compared to the PVA-gel, rising from 330.6 °C to 344.4 °C. Furthermore, in the DTG curves of the PU\PVA-Li0 DN hydrogel (Figure 1d), the maximum degradation temperature at 365.8 °C was due to the thermal degradation of PU and PVA chains, which is higher than that of PVA-gel (363.4 °C). This finding provides direct evidence that the PU\PVA-Li0 hydrogel with a double network structure exhibits better thermal stability than the PVA-gel with a single network. This phenomenon can likely be ascribed to the strong interactions (hydrogen-bonding, π–π stacking, and hydrophobic interactions) between the chains of PU and PVA, leading to the formation of a rigid network and a higher crosslinking degree of the PU\PVA-Li0 DN hydrogel than that of PVA-gel [33,34].

**Figure 2:**
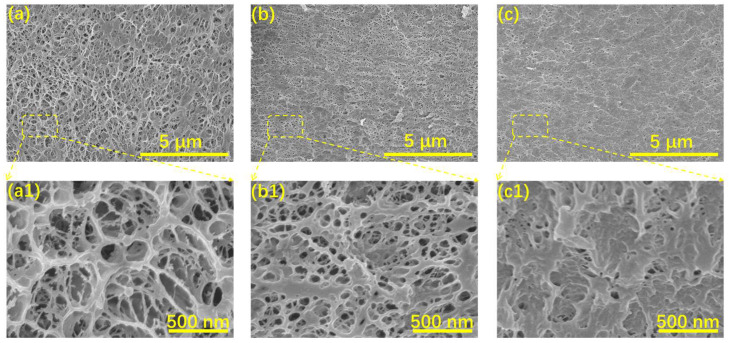
SEM images for PVA-gel (**a**,**a1**), PU/PVA-Li0 (**b**,**b2**), and PU/PVA-Li0.7 (**c**,**c1**).

The microstructure is considered to play a crucial role in determining the mechanical strength and toughness of PU/PVA-Li hydrogels. Thus, the microscopic structure of PVA-gel, PU/PVA-Li0, and PU/PVA-Li0.7 were analyzed using SEM, as depicted in Figure 2. The PVA-gel exhibited loose microstructures and large pore size (Figure 2a,a1), which make it typically brittle and susceptible to fracture, resulting in a restricted capacity for energy dissipation. With the introduction of a PU three-dimensional network into PVA-gel, PU/PVA-Li0 exhibited a more compact network structure with fewer pores than that of the PVA-gel because of the strong H-bonding interactions between PU and PVA (Figure 2b,b1). Moreover, after soaking in the LiCl solutions, the resultant PU/PVA-Li0.7 hydrogel exhibited a highly compact structure (Figure 2c,c1), indicating an increased crosslinking density. The incorporation of LiCl has the potential to induce polymer chain contraction through ionic coordination, thus promoting the formation of hydrophobic associations within the hydrogel matrixes [24,35].

### 3.2. Mechanical Properties of PU/PVA-Li Hydrogels

**Figure 3:**
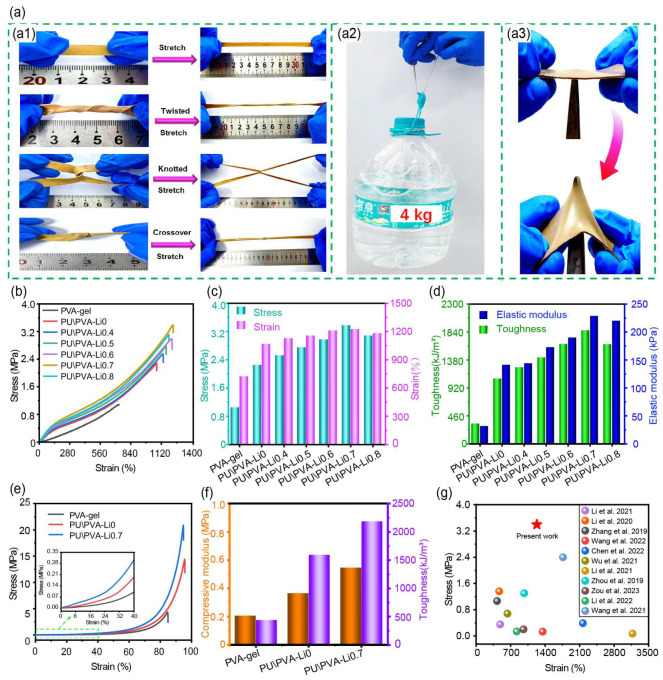
(**a**) Photographs showing the outstanding mechanical performances of PU/PVA-Li0.7 hydrogel: stretching, twisting, knotting, and crossover stretching (**a1**); the PU/PVA-Li0.7 hydrogel, with dimensions of 10 mm in width and 1.2 mm in thickness, successfully lifted a 4-kg weight of water (**a2**); PU/PVA-Li0.7 subjected to puncture by sharp scissors (**a3**). Tensile stress–strain curves of the PU/PVA-Li hydrogels (**b**); the corresponding stress and strain (**c**) and elastic modulus and toughness (**d**) of the PU/PVA-Li hydrogels. Compressive stress–strain curves of the PU/PVA-Li hydrogels (**e**); the corresponding compressive modulus and toughness of the PU/PVA-Li hydrogels (**f**). (**g**) The tensile strength and fracture elongation of the PU/PVA-Li hydrogels were compared with those of other ionic conductive hydrogels [20,24,36,37,38,39,40,41,42,43,44].

Owing to the synergistic effect of inter/intra-molecular hydrogen bonding, hydrophobic associations, π–π stacking, and the interpenetrating network structure of interwoven polymer chains, the obtained PU/PVA-Li hydrogel demonstrated outstanding mechanical properties. For visual evidence, several mechanical experiments were conducted, as displayed in Figure 3a. The PU/PVA-Li0.7 hydrogel could be readily stretched, twisted, knotted, and crossover stretched to large deformations, demonstrating high stretchability and ductility (Figure 3(a1)). In addition, PU/PVA-Li0.7 strips could readily lift a weight of 4 kg water, revealing outstanding mechanical strength (Figure 3(a2)). More importantly, as shown in Figure 3(a3), PU/PVA-Li0.7 did not receive any punctures from sharp scissors, indicating that the hydrogel possessed excellent penetration resistance.

Considering the diverse mechanical loads that PU/PVA-Li hydrogels may encounter during practical applications, both tensile and compression tests were conducted on the hydrogels. As exhibited in Figure 3b–f, the PVA-gel demonstrated ductile behavior with a fracture strain of 731.3% and a low tensile strength of 1.1 MPa, as well as a fracture strain of 84.8% and a small compressive strength of 4.3 MPa. After incorporation of the PU three-dimensional network, the tensile stress and fracture strain of the PU/PVA-Li0 displayed a dramatic increase, reaching 2.3 MPa and 1075.7%, respectively. The corresponding elastic modulus and toughness showed remarkable enhancements, measuring 141.4 kPa and 1074.9 kJ/m^3^, respectively. These values were found to be 44 and 3 times greater than those observed in the PVA-gel. Furthermore, the compressive stress of the PU/PVA-Li0 hydrogel could reach up to 14.4 MPa at a strain of 95.7%, resulting in a significant increase of 176% in the compression modulus and 356% in the toughness compared to those of the PVA-gel (Figure 3e,f). This result suggests that the introduction of the 3D chemical crosslinking network of PU led to a substantial improvement in the mechanical strength of the PVA-gel. Interestingly, incorporating LiCl into the hydrogel matrix could further enhance the mechanical properties of the PU/PVA-Li0 hydrogel. As depicted in Figure 3b,d, as the LiCl concentration increased from 0 to 0.7 M, the tensile strength and fracture strain of the hydrogel improved from 2.3 MPa to 3.4 MPa and from 1075.7% to 1231.8%, respectively. The corresponding elastic modulus and toughness of PU/PVA-Li0.7 reached up to 228.9 kPa and 1868.6 kJ/m^3^, indicating that the introduction of an appropriate concentration of LiCl further enhanced the mechanical properties of the PU/PVA-Li0 hydrogel. The reason for the above results was inferred to be that the introduction of LiCl facilitates the contraction of polymer chains via ionic coordination [24,45]. This process, in turn, promotes the formation of physical hydrophobic interactions among the PU chains and increases the crosslinking density [35]. As a result, the mechanical properties of the PU/PVA-Li hydrogel are significantly improved. However, when the LiCl concentration increased to 0.8 M, there was a slight decrease in both the tensile strength and fracture strain, which may be attributed to the excessive content of the LiCl rupturing the inter-molecular hydrogen bonds in the PU/PVA-Li network [46]. Similarly, PU/PVA-Li0.7 exhibited an enhanced compression strength after loading LiCl, and a compressive stress of 20.9 MPa at a fracture strain of 94.7% with a compression toughness of 2196.4 kJ/m^3^ was achieved (Figure 3e,f). Moreover, Figure 3g provides a comparison chart of the mechanical properties of the PU/PVA-Li hydrogels with those of other ionic conductive hydrogels. The majority of ionic conductive hydrogels typically demonstrate either high mechanical strength or large fracture strain but not both simultaneously. Impressively, our PU/PVA-Li hydrogels achieved a remarkable balance of high strength and excellent elongation, rendering them highly suitable for diverse applications in flexible electronic devices.

**Figure 4:**
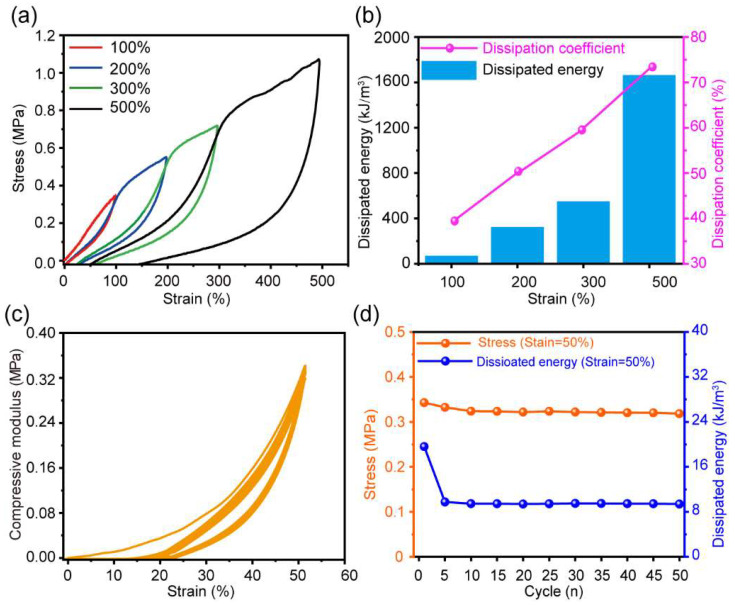
(**a**,**b**) Cyclic tensile loading–unloading curves and their corresponding dissipated energy and energy dissipation coefficients of PU/PVA-Li0.7 at strains of 100%, 200%, 300%, and 500%. (**c**) Stress–strain curves of PU/PVA-Li0.7 hydrogel for 50 consecutive compression-relaxation cycles. (**d**) The maximal stress and corresponding dissipated energy of PU/PVA-Li0.7 hydrogel for every five cycles in 50 consecutive compression-relaxation cycles.

Moreover, the combination of hydrophobic conjugation, inter/intra-molecular hydrogen bonding, and ionic coordination within PU/PVA-Li hydrogels synergistically enhances their mechanical strength and substantially improves the hydrogel’s toughness through effective energy dissipation. The energy dissipation capacity and coefficient of the PU/PVA-Li hydrogels (specifically PU/PVA-Li0.7) were further investigated through cyclic tensile tests. The notable hysteresis observed in Figure 4a suggests that the PU/PVA-Li0.7 hydrogel possesses the ability to effectively dissipate energy. Moreover, the hysteresis energy demonstrates a sharp increase as the strain increases, which is a crucial characteristic of tough hydrogels. The quantified results, as shown in Figure 4b, demonstrate a significant increase in the energy dissipation of the PU/PVA-Li0.7 hydrogel from 71.1 kJ/m^3^ at a strain of 100% to 1682.9 kJ/m^3^ at a strain of 500%, accompanied by a corresponding dissipation coefficient reaching up to 73.8%. Additionally, a series of 50 continuous compression–relaxation cycles at a strain of 50% was performed to assess the fatigue-resistance properties of the PU/PVA-Li hydrogel. As depicted in Figure 4c, the PU/PVA-Li0.7 hydrogel dissipated a substantial amount of energy during the first cycle, and then displayed similar hysteresis energy for 2nd–50th cycles. Figure 4d summarizes the maximum stress and dissipated energy at intervals of five cycles. The maximum stress of PU/PVA-Li0.7 hydrogel remained nearly constant during 50 consecutive compressive-relaxation cycles, indicating that the dual-network hydrogel exhibits excellent elasticity and good fatigue resistance. Additionally, despite a decrease in the dissipated energy after undergoing 50 cycles, it remained greater than 9.4 kJ/m^3^ (equivalent to 95.9% of the second cycle). The above results indicated that the hydrophobic conjugation, inter/intra-molecular hydrogen bonding, and ionic coordination within PU/PVA-Li hydrogels were robust enough to act as distinctive dynamic junction points within the hydrogel matrix. Under external loading, the dynamic junction points, such as hydrophobic conjugation, inter/intra-molecular hydrogen bonding, and ionic coordination, within the PU/PVA-Li hydrogel act as sacrificial bonds, dissipating energy to prevent fracture. Meanwhile, the chemical crosslinked PU 3D networks maintain the configuration of the PU/PVA-Li hydrogels without fracturing. Once the external loading was removed, the temporarily fractured dynamic junction points within the PU/PVA-Li0.7 hydrogel could reassemble, providing the hydrogel with excellent fatigue-resistance properties. The above remarkable resilience and fatigue resistance of PU/PVA-Li0.7 hydrogel are of significant importance in the fields flexible sensors and soft robots.

### 3.3. Electronic Behavior, Sensing Performance, and Applications of the PU/PVA-Li Hydrogels

**Figure 5:**
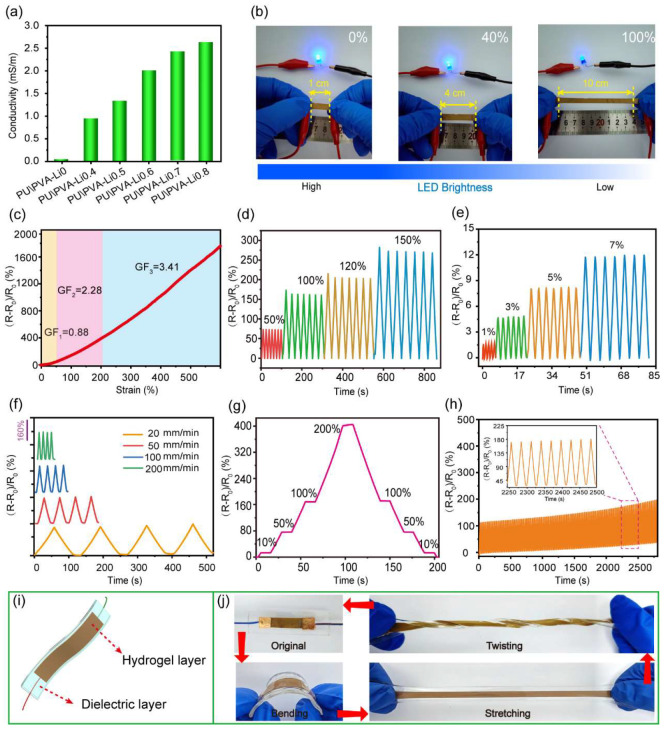
(**a**) The ionic conductivity of PU/PVA-Li hydrogels with different LiCl concentrations. (**b**) Luminance change of an LED bulb with the elongation of the PU/PVA-Li0.7 hydrogel. (**c**) The (R − R_0_)/R_0_ value of the PU/PVA-Li0.7 hydrogel changes with the applied strain. Relative resistance changes under small (**d**) and large (**e**) strains. (**f**) The (R − R_0_)/R_0_ variations under cyclic stretching-releasing at 50% strain at stretching speeds of 20, 50, 100, and 200 mm/min. (**g**) The (R − R_0_)/R_0_ variation under various applied strains (10%, 50%, 100%, and 200%) for a period of 10 s. (**h**) The (R − R_0_)/R_0_ response during repeated 100 stretching–releasing cycles (50% strain). (**i**) Assembly mechanism diagram of the PU/PVA-Li0.7 hydrogel sensor. (**j**) Outstanding flexibility, stretchability, and elastic recovery ability of the PU/PVA-Li0.7 hydrogel sensor.

The presence of Li^+^ and Cl^−^ ions within the hydrogel not only enhances its mechanical performance but also plays a crucial role in conferring exceptional ionic conductivity to the PU/PVA-Li hydrogel via ionic migration. This property is essential for the practical application of conductive hydrogels in wearable electronics. As shown in Figure 5a, the ionic conductivity of PU/PVA-Li hydrogels increased from 0.02 to 2.62 mS/m upon increasing the LiCl concentration from 0 to 0.8 M. This outcome is due to the augmented ion movement in the hydrogel network under a high LiCl concentration. Moreover, when the hydrogel (PU/PVA-Li0.7) was connected to the channel of an LED bulb (Figure 5b), the luminance synchronously changed with the variation of stretching strain, indicating the excellent electrical sensitivity of the hydrogel. During stretching deformation, the hydrogel experiences an increase in stretching deformation, leading to a reduction in the ion channel and an elongation of the migration path. Consequently, this process results in relatively slower migration of Li^+^ and Cl^−^ ions within the hydrogel. Therefore, as depicted in Figure 5c, the PU/PVA-Li0.7 hydrogel displays a clear strain-dependent responsiveness in terms of the relative resistance change ((R − R_0_)/R_0_, where R_0_ and R are the resistance of the hydrogel at 0% strain and applied strain, respectively). The electrical sensitivity of the PU/PVA-Li0.7 hydrogel is evaluated by calculating the gauge factor (GF) using the slope of (R − R_0_)/R_0_ versus the strain curve. As shown in Figure 5c, the (R − R_0_)/R_0_ value increased with increasing stretching strain, and the corresponding GF was divided into three linear responsive areas, namely 0–50% with a GF of 0.88, 50–200% with a GF of 2.28, and 200–600% with a GF of 3.41, indicating the high strain sensitivity and wide detectable range of the PU/PVA-Li0.7 hydrogel.

To evaluate the PU/PVA-Li hydrogel’s potential for use in wearable electronic devices, a flexible strain sensor was created by sandwiching a PU/PVA-Li0.7 hydrogel between two VHB tapes (4905, 3 M), as depicted in Figure 5i. The obtained PU/PVA-Li0.7 hydrogel sensor exhibited significant stretchability, flexibility, and elastic recovery ability (Figure 5j). Figure 5d shows the relative resistance of the PU/PVA-Li0.7 hydrogel sensor during stretching/releasing cycling at strains of 50%, 100%, 120%, and 150%, and the corresponding (R − R_0_)/R_0_ values were 72.3%, 161.4%, 204.7%, and 273.8%, respectively, indicating the excellent stability and repeatability of the sensor device. In particular, as depicted in Figure 5e, our PU/PVA-Li0.7 hydrogel sensor demonstrated high sensitivity even to small strains ranging from 1% to 7%. Moreover, the PU/PVA-Li0.7 hydrogel sensor could accurately respond to stretching/releasing cycling under diverse tensile speeds, ranging from 20 to 200 mm/min at a strain of 100%. This result highlights its potential for detecting dynamic forces effectively. Figure 5g illustrates the change of (R − R_0_)/R_0_ caused by the step increase/decrease in stretching strain. The (R − R_0_)/R_0_ value increased to different levels with each step increase in stretching strain from 10% to 200%. It promptly reverted to its original level upon the stepwise release of the stretching strain. This behavior suggests the exceptional sensing capabilities of the PU/PVA-Li0.7 hydrogel sensor. In particular, (R − R_0_)/R_0_ remained constant when the stretching strain was maintained at a certain value, demonstrating the excellent electrical stability of our hydrogel sensor. Therefore, by controlling the stretching strain, the real-time (R − R_0_)/R_0_ change can be accurately monitored, allowing for precise and dynamic tracking of the hydrogel sensor’s electrical response. Importantly, as shown in Figure 5h, our PU/PVA-Li0.7 hydrogel sensor exhibited a stable response in the electrical signal over 100 successive stretching–releasing cycles at strain of 50%, further indicating the excellent stability and reliability of the hydrogel sensor. This stable electrical signal response performance was attributed to the remarkable dynamic reconfiguration property within the PU/PVA-Li0.7 hydrogel matrix.

**Figure 6:**
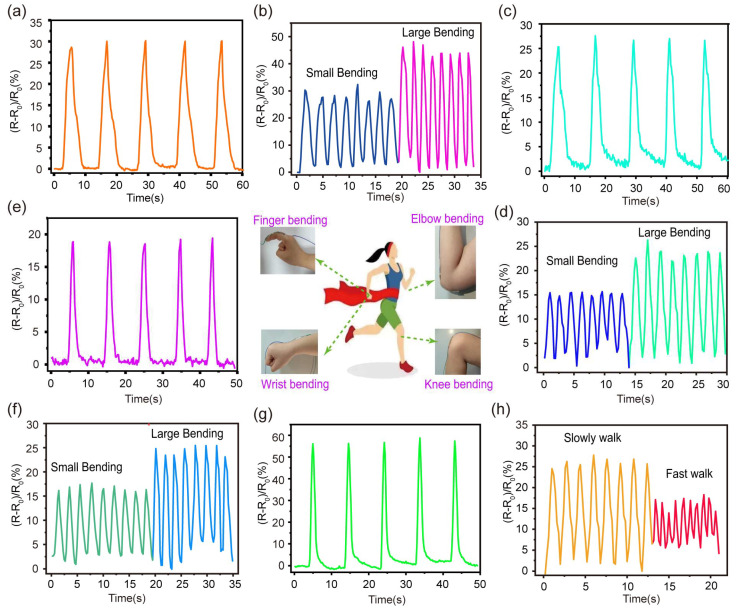
Responsiveness of PU/PVA-Li hydrogel sensor to various human motions. The flexion movements of finger (**a**), elbow (**c**), wrist (**e**), and knee joint (**g**) at certain angles. The flexion movements of finger (**b**), elbow (**d**), and wrist (**f**) under different angles. (**h**) Signal from the knee joint with different walking frequencies.

Based on its outstanding stretchability, extremely wide detection range, and high sensitivity, the PU/PVA-Li hydrogel sensor can be effectively utilized as a wearable electronic device for monitoring various joint movements of human motions. As shown in Figure 6a,c,e,g, the (R − R_0_)/R_0_ change was uniform and repeatable when the sensor was mounted on the finger, elbow, wrist, and knee joint bent at a certain angle. Specifically, (R − R_0_)/R_0_ exhibited a rapid increase when the joints were bent and fully recovered to the original value upon straightening, suggesting the quick response and excellent self-recovery capabilities of the PU/PVA-Li hydrogel sensor. On the other hand, the joint (finger, elbow, and wrist) bending angle could be reflected by monitoring the intensity of the relative (R − R_0_)/R_0_ value (Figure 6b,d,f). Moreover, variations in the relative resistance signal waveforms were observed during knee joint flexion at different walking frequencies, including both slow and fast walking speeds (Figure 6g).

**Figure 7:**
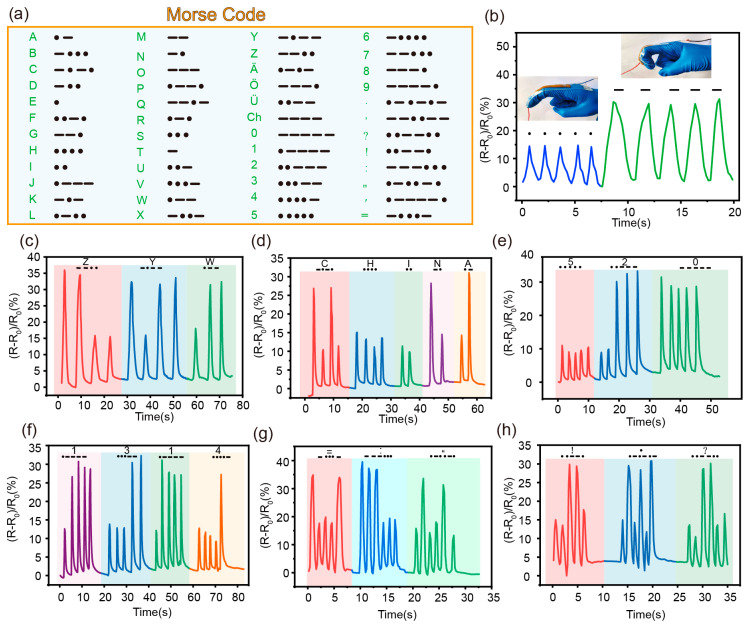
(**a**) Schematic diagram of Morse code in English letters, numbers, and symbols. (**b**) The relative resistance changes when different degrees of finger bending were used to record “dots” and “dashes” of Morse code. PU/PVA-Li hydrogel sensor can accurately record English letters (“ZYW”, “CHINA”) (**c**,**d**), numbers (“520”, “1314”) (**e**,**f**), and punctuation marks (= :”, ! ·?) (**g**,**h**) through Morse code.

Our PU/PVA-Li0.7 hydrogel sensor with high sensitivity, excellent stability and reliability presents significant potential for applications in information encryption and decryption, offering enhanced security measures. Morse code, consisting of “dots” and “dashes”, is a time-honored and efficient method of communication that represents different letters, numbers, and punctuation marks through distinct combinations of these two elements (Figure 7a). As shown in Figure 7b, the (R − R_0_)/R_0_ change of the PU/PVA-Li0.7 hydrogel sensor induced by small finger bending was designated as a “dot”, while large finger bending was designated as a “dash”. By bending the PU/PVA-Li0.7 hydrogel sensor, the desired message could be accurately transmitted through changes in the sensor resistance in accordance with the Morse code table, for example, “ZYW” and “CHINA” (Figure 7c,d). The messages “520” and “1314” also could be output in this way (Figure 7e,f). Additionally, expression of punctuation marks, such as “= :”” and “! · ?”, were also conducted (Figure 7g,h). Therefore, our PU/PVA-Li0.7 hydrogel sensor could be used for information recording and transmission, offering new possibilities for the application of hydrogel sensors. Thus, the PU/PVA-Li hydrogel developed in this study demonstrates significant potential for information recording and transmission, thereby broadening the range of applications for hydrogel sensors.

## 4. Conclusions

In summary, a PU/PVA-Li ionic DN hydrogel, comprising a covalently crosslinked PU 3D network and a physically crosslinked PVA network, was successfully fabricated, followed by the incorporation of LiCl salts into the hydrogel matrix. The obtained PU/PVA-Li double-network hydrogel demonstrated exceptional mechanical properties, including high strength (3.4 MPa), remarkable toughness (1868.6 kJ/m^3^), and high compressive strength (up to 20.9 MPa). During deformation, the inter/intra-molecular hydrogen bonding, hydrophobic associations, π–π stacking, and interpenetrating network structure of PU and PVA chains were first disrupted. This mechanism effectively dissipates energy and prevents the fracturing of the main networks, resulting in the remarkable fatigue resistance and good resilience of the PU/PVA-Li hydrogels. More notably, the incorporation of LiCl not only can further optimize the mechanical strength of the PU/PVA-Li hydrogels but also can impart to them exceptional ionic conductivity (2.62 mS/m) to realize high sensitivity (GF = 3.41 at strain 200–600%) to mechanical strain over a wide range. Consequently, the PU/PVA-Li hydrogel was successful in its application for a flexible electronic device for monitoring f various joint movements and information encryption/decryption using Morse code. This work provides a facile strategy to create an ionic conductive double-network hydrogel with exceptional strength and toughness utilizing ecofriendly natural extracts and biocompatible polymers. The developed PU/PVA-Li hydrogel could serve as a versatile platform for manufacturing the next generation of high-performance flexible wearable electronics with multifunctional capabilities.

## Data Availability

The data presented in this study are available on request from the corresponding author.
